# Dismantling darkness: interdisciplinary perspectives on melanin degradation

**DOI:** 10.1007/s11274-026-04995-x

**Published:** 2026-05-02

**Authors:** Lucas Martins Alcântara, Camilla de Oliveira Alves, Elisa Gonçalves Medeiros, Deborah Santos Cintra, Kamilla Xavier Gomes, Yasmim Cristina de Almeida Lima, Susana Frases, Marcia Ribeiro Pinto, Radamés JB Cordero, Allan Jefferson Guimarães

**Affiliations:** 1 Laboratório de Bioquímica e Imunologia das Micoses, Instituto Biomédico, Fluminense Federal University, Niterói, Brazil; 2 Programa de Pós-Graduação em Microbiologia e Parasitologia Aplicadas, Instituto Biomédico, Fluminense Federal University, Niterói, Brazil; 3 Pós-Graduação em Imunologia e Inflamação, Instituto de Microbiologia Paulo de Góes, Federal University of Rio de Janeiro, Rio de Janeiro, Brazil; 4 Laboratório de Biologia de Fungos, Instituto de Biofísica Carlos Chagas Filho, Federal University of Rio de Janeiro, Rio de Janeiro, Brazil; 5 Rede Micologia RJ – Fundação de Amparo à Pesquisa do Estado do Rio de Janeiro (FAPERJ), Rio de Janeiro, Brazil; 6 Department of Molecular Microbiology and Immunology, Johns Hopkins University, Baltimore, USA; 7 National Institute of Science and Technology (INCT) in Human Pathogenic Fungi, São Paulo, Brazil; 8 Depto. de Microbiologia e Parasitologia – MIP, Instituto Biomédico, Universidade Federal Fluminense, Rua Prof. Hernani Pires de Melo 101, São Domingos, Niterói, Rio de Janeiro, 24210-130 Brazil

**Keywords:** Microbial Melanin, Degradation, Laccase, Lignin Peroxidase, Manganese Peroxidase, Bioremediation, DHN-melanin

## Abstract

Microbial melanins are exceptionally recalcitrant biopolymers that facilitate fungal virulence and biodeterioration. This review synthesizes physical, chemical, and biological degradation strategies for major microbial melanin categories, aligning technical mechanisms with their translational potential. While physical methods such as ultrasound-assisted (UAE) and microwave-assisted extraction (MAE) function as matrix-disrupting pretreatments rather than standalone degradation strategies, chemical approaches remain the analytical standard for structural fingerprinting via specific markers like pyrrole-2,3,5-tricarboxylic acid (PTCA), pyrrole-2,3-dicarboxylic acid (PDCA) and 4-amino-3-hydroxyphenylalanine (AHP). Conversely, biological approaches, primarily utilizing ligninolytic enzymes such as laccases and peroxidases that catalyze the oxidative breakdown of complex melanin structures, offer the most sustainable route for applied degradation. We critically evaluate emerging biotechnological solutions, including immobilized nanobiocatalysts like LiP@MFO-GO (LiP immobilized onto graphene oxide coated magnetic nanoparticles), engineered synergistic laccase-peroxidase complexes for in situ H_2_O_2_ recycling, and the use of glutathione peroxidase (GPX) from lysosome-related extracts, highlighting their stability and reusability under industrial conditions. Finally, we outline a strategic framework to overcome mediator toxicity and ensure the generation of biocompatible fragments, providing a roadmap for innovative applications in cosmetics, food preservation, and environmental remediation.

## Introduction

Melanins comprise a diverse group of large, heterogeneous, and high molecular-weight pigments, typically dark-brown to black, synthesized by organisms across all biological kingdoms (Bell and Wheeler 1986; Nosanchuk et al. 2015; Cordero and Casadevall 2017; Chhoker et al. 2025). In microorganisms, particularly fungi, these biopolymers are not essential for primary metabolism and growth, but provide substantial ecological and physiological advantages and significant survival benefits (Bell and Wheeler 1986; Chhoker et al. 2025). Fungal melanins are also well-recognized key virulence factors in several pathogenic species, contributing to resistance against host defenses and antifungal agents (Nosanchuk et al. 2015; Cordero and Casadevall 2017; Cordero 2017).

In fungi, melanin is primarily found in the cell wall, where it is covalently anchored to chitin and glucans, reinforcing the structural matrix and forming a robust protective barrier (Bell and Wheeler 1986; Eisenman and Casadevall 2012a; Nosanchuk et al. 2015; Cordero and Casadevall 2017; Chhoker et al. 2025). The melanin structure confers resistance to various environmental stressors, including ultraviolet (UV) and ionizing radiation (Bell and Wheeler 1986; Cordero and Casadevall 2017; Muñoz-Torres et al. 2024; Chhoker et al. 2025), desiccation (Bell and Wheeler 1986; Cordero and Casadevall 2017; Chhoker et al. 2025), and extreme temperatures (Cordero and Casadevall 2017; Guo et al. 2023; Chhoker et al. 2025). Functionally, melanins act as potent antioxidants, by scavenging/quenching free radicals and stabilizing the cellular redox balance (Cordero and Casadevall 2017; Muñoz-Torres et al. 2024; Subramaniam et al. 2024; Chhoker et al. 2025). Additionally, melanin physically shields the cell wall from enzymatic lysis by sterically hindering the access of hydrolytic enzymes, such as chitinase and glucanase, to structurally important polysaccharides on the fungal cell wall (Bloomfield and Alexander 1967; Luther and Lipke 1980; Bell and Wheeler 1986; Eisenman and Casadevall 2012a; Cordero and Casadevall 2017).

Despite its biological advantages for fungi, melanin’s high chemical recalcitrance represents a significant challenge to degradation (Muñoz-Torres et al. 2024). Regarding its structure, melanin shares notable compositional similarities with lignin, being a complex, irregular, and cross-linked aromatic polymer, composed of indolic, phenolic, and quinonoid subunits that undergo extensive oxidative polymerization (Tavzes et al. 2009; Sung et al. 2019; Rathour et al. 2024). Overall, melanin’s structural complexity confers exceptional chemical and thermal stability, contributing to the resistance of soil organic matter to rapid degradation, making melanin one of the most durable biopolymers (Luther and Lipke 1980; Bell and Wheeler 1986). Its persistent nature has broad implications – from the dark pigmented fungal stains caused by melanized fungi, causing aesthetic and structural issues, such as on paper-based cultural heritage materials (Tavzes et al. 2009), to potential alterations in oxidative processes in lipid-rich foods, such as pork lard (Subramaniam et al. 2024).

In humans, excessive eumelanin production is linked to the development of skin hyperpigmentation disorders, such as melasma and post-inflammatory pigmentation (Sadaqat et al. 2020; Jeon et al. 2021). Current depigmenting agents including kojic acid, hydroquinone, and arbutin primarily act by inhibiting tyrosinase, a key phenoloxidase and rate-limiting enzyme of the melanogenic pathway (Shin et al. 2019; Sadaqat et al. 2020; Jeon et al. 2021). However, these compounds generally do not degrade pre-existing melanin and may exhibit undesirable side effects, including cytotoxicity, skin irritation, and ochronosis, as well as potential carcinogenic risks, emphasizing the need for safer alternatives (Simon and Peles 2010; Shin et al. 2019; Sadaqat et al. 2020). While human melanin is the primary target for cosmetic depigmenting agents, microbial melanins represent the most chemically recalcitrant forms of these pigments and are often covalently anchored to cell wall polysaccharides. Understanding the dismantling of these robust microbial structures provides a fundamental framework for developing highly efficient and safe enzymatic whitening technologies that are translatable to human skincare (Eisenman and Casadevall 2012a; Pillaiyar et al. 2017).

Consequently, effective melanin polymer degradation or decolorization strategies are increasingly sought after for diverse safer applications, including cosmetics (Eisenman and Casadevall 2012b; Kim et al. 2016a; Shin et al. 2019; Sadaqat et al. 2020), art restoration and conservation (Tavzes et al. 2009), food preservation (Subramaniam et al. 2024), and the bioremediation of dye-contaminated effluents, including industrial waste (Muñoz-Torres et al. 2024). Despite the abundance of studies addressing melanin biosynthesis, the mechanisms and technologies underlying melanin degradation remain fragmented and underexplored.

The imperative for microbial melanin degradation stems from its role as a persistent environmental and industrial pollutant. In cultural heritage conservation, 1,8-dihydroxynaphthalene (DHN)-melanized fungi are major colonizers that form chemically stable deposits, leading to irreversible structural and aesthetic damage to paper, wood, and parchment. In the food industry, the presence of melanin-producing fungi in lipid-rich products can accelerate oxidative rancidification, compromising shelf life and safety. Furthermore, the discharge of industrial effluents containing melanin-like pigments necessitates efficient removal strategies, as these polymers resist conventional biological wastewater treatments. Thus, dismantling the melanin polymer is not merely a biochemical challenge but a prerequisite for sustainable industrial processes and heritage preservation.

**Fig. 1:**
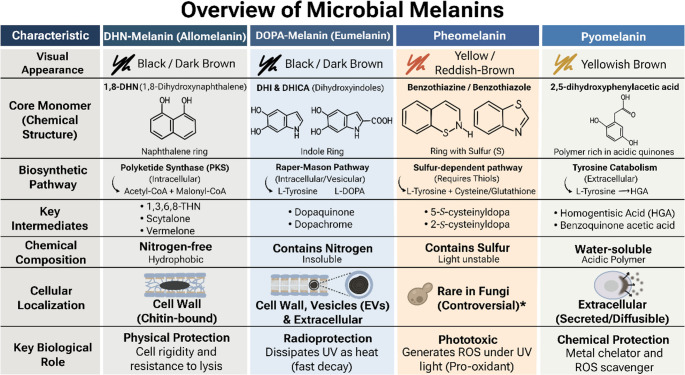
Comparative overview of the primary microbial melanin types. The image summarizes the visual characteristics, biosynthetic pathways, key biochemical intermediates, and biological functions of DHN-melanin, DOPA-melanin, Pheomelanin, and Pyomelanin. Distinctions are highlighted based on core monomer structures, nitrogen/sulfur composition, and cellular localization within the microbial cell wall or extracellular space. *Note: The synthesis of pheomelanin by fungi is considered controversial; while some studies suggest its presence in specific taxa, robust biochemical characterization remains scarce compared to other fungal melanins

This review compiles and analyzes the current knowledge concerning the melanin degradation mechanisms, catalysts, applications and main challenges faced for the existing methods, with emphasis on microbial and enzymatic processes and their integration into physical and chemical degradation systems. Understanding the structural diversity of microbial melanins such as L-3,4-dihydroxyphenylalanine (L-DOPA) melanin, DHN-melanin, and pyomelanin is fundamental, as their distinct chemical architectures determine resistance to oxidative or enzymatic breakdown (Bell and Wheeler 1986; Wakamatsu and Ito 2023). The structural diversity, biosynthetic pathways, and biological functions of these primary microbial melanin types are summarized in Fig. 1. By consolidating recent advances, this work aims to outline the state-of-the-art in melanin degradation research, identify knowledge gaps, and propose directions for future biotechnological exploitation of these strategies.

## Types of fungal melanin and their synthetic pathways

### Synthesis and functional diversity of fungal melanins

#### DHN-melanin (Allomelanin)

The 1,8-dihydroxynaphthalene (DHN)-melanin, also classified as allomelanin, is the characteristic dark hydrophobic pigment deposited in the cell walls of numerous filamentous fungi, predominantly within the *Ascomycotina* and *Deuteromycotina* phyla (Bell and Wheeler 1986; Eisenman and Casadevall 2012b; Guo et al. 2023; Chhoker et al. 2025). Unlike mammalian eumelanin, which is derived from L-DOPA, DHN-melanin is synthesized intracellularly via the polyketide synthase (PKS) pathway. This biosynthetic process utilizes acetyl-CoA and malonyl-CoA as precursors to form 1,3,6,8-tetrahydroxynaphthalene (1,3,6,8-THN), which subsequently undergoes sequential reduction and dehydration steps culminating in polymerization within the cell wall (Bell and Wheeler 1986; Eisenman and Casadevall 2012b; Chhoker et al. 2025).

#### Biosynthesis of DHN-melanin

The DHN-melanin is categorized as an allomelanin, meaning it is a nitrogen-free polymer produced via the polyketide synthase (PKS)-dependent pathway (Pralea et al. 2019; Singh et al. 2021; Guo et al. 2023; Muñoz-Torres et al. 2024). Its biosynthetic pathways utilize acetate or malonyl-CoA units as building blocks in iterative condensation reactions catalyzed by PKS enzymes, encoded by genes such as Alb1/PksP in *Aspergillus fumigatus* and their orthologues in other fungi (Bell and Wheeler 1986; Eisenman and Casadevall 2012b; Singh et al. 2021; Muñoz-Torres et al. 2024; Chhoker et al. 2025). The first stable intermediate formed is 1,3,6,8-tetrahydroxynaphthalene (1,3,6,8-THN), which subsequently undergoes a cascade of reduction and dehydration steps (Bell and Wheeler 1986; Tsai et al. 1999; Eisenman and Casadevall 2012a; Singh et al. 2021; Muñoz-Torres et al. 2024; Chhoker et al. 2025). Initially, 1,3,6,8-THN is reduced to scytalone (by reductases, such as the *Arp2*); scytalone is sequentially dehydrated to 1,3,8-trihydroxynaphthalene (1,3,8-THN) (by a dehydratase, such as *Arp1*); 1,3,8-THN is reduced to vermelone (also catalyzed by *Arp2*); and vermelone is dehydrated to the final monomer product, 1,8-dihydroxynaphthalene (1,8-DHN) (by a dehydratase, such as *Abr1*) (Bell and Wheeler 1986; Singh et al. 2021; Chhoker et al. 2025). The terminal oxidative polymerization of 1,8-DHN monomer into the allomelanin polymer is mediated by surface-associated laccase/phenoloxidases, such as *Abr2* (Bell and Wheeler 1986; Eisenman and Casadevall 2012b; Chhoker et al. 2025). In *A. fumigatus*, these DHN-melanin biosynthetic pathway genes are organized into a coordinately regulated gene cluster that includes *alb1* (pksP), *arp1*, *arp2*, *abr1*, and *abr2* (Tsai et al. 1999). The expression of these genes is developmentally regulated, being low during vegetative growth but strongly upregulated during conidiation, particularly in regions of aerial hyphae, areas of the vesicles, where phialides would emerge, and nascent conidia, where Abr1 and Abr2 localize to the outer wall (Upadhyay et al. 2013). Environmental conditions, such as copper availability, modulate DHN-melanin production. Under copper-limited conditions, the expression of a CtpA copper transporter was crucial for proper melanization, driving the upregulated expression of *abr1* and *abr2* (Upadhyay et al. 2013). Overall, this regulatory plasticity underscores the DHN-melanin’s role in maintaining the redox balance and protecting against stress.

#### Distribution of DHN-melanin and biological roles

DHN-melanin is widespread throughout the fungal kingdom, including numerous saprophytic and pathogenic species, such as *A. fumigatus*, *Alternaria alternata*, *F. pedrosoi*, *Cladosporium cladosporioides*, *Wangiella dermatitidis*, *Exophiala dermatitidis*, *Magnaporthe oryzae*, *Colletotrichum lagenarium* and *Bipolaris oryzae* (Tsai et al. 1999; Woo et al. 2004; Singh et al. 2021; Koehler et al. 2024). Across these taxa, DHN-melanin contributes to cell wall rigidity, resistance to UV radiation, protection against desiccation and oxidants, and resistance to phagocytosis or antifungal agents. In pathogenic fungi, such as *A. fumigatus*, *Exophiala*, and *Wangiella*, DHN-melanin masks pathogen-associated molecular patterns (PAMPs), thereby evading immune recognition and dampening host inflammatory responses (Eisenman and Casadevall 2012b; Cordero and Casadevall 2017).

The DHN-melanin pathway can be specifically inhibited by tricyclazole [5-methyl-1,2,4-triazolo(3,4-b)benzothiazole], which blocks the reductive steps catalyzed by hydroxynaphthalene reductases (Arp1/Arp2), preventing the conversion of 1,3,6,8-THN to scytalone and vermelone. This leads to the accumulation of reddish or orange intermediates, resulting in albino phenotypes (Woo et al. 2004; Cunha et al. 2005, 2010). In *M. oryzae* and *C. lagenarium*, tricyclazole-treated strains exhibit defective appressorial melanization, resulting in a loss of turgor pressure and failure to penetrate host surfaces, demonstrating the critical role of DHN-melanin in mechanical strength and virulence (Kunova et al. 2013).

The deposition of this polymer confers structural integrity and protection against environmental and immunological stressors in various pathogenic and environmental fungi described in the analyzed literature:

- *Fonsecaea pedrosoi*: In this etiological agent of chromoblastomycosis, DHN-melanin acts as an essential virulence factor, accumulating in the cell walls of conidia and, most notably, in sclerotic (muriform) cells (Franzen et al. 2006). Research has demonstrated that this melanin functions as an antioxidant “trap,” effectively shielding the fungus from oxidative destruction mediated by hydrogen peroxide (H2O2), nitric oxide (NO), and hypochlorite (HOCl) produced by macrophages and neutrophils during phagocytosis (Cunha et al. 2005, 2010; Franzen et al. 2006).
- *Pyricularia oryzae* (syn. *Magnaporthe grisea*): In phytopathogens, DHN-melanin is fundamental to pathogenesis, not for vegetative growth, but for the mechanics of infection. It provides the necessary rigidity and impermeability to appressorial walls, allowing the accumulation of the turgor pressure essential for the mechanical penetration of the host plant epidermis (Bell and Wheeler 1986; Chhoker et al. 2025).
- *Aspergillus fumigatus* and *Wangiella dermatitidis*: In these pathogens, DHN-melanin contributes to virulence and survival by conferring resistance to cellular lysis and masking the pathogen from the host immune system (Eisenman and Casadevall 2012b; Chhoker et al. 2025).
- *Pestalotiopsis* sp.: This genus is highlighted as a high-yield producer of melanin, possessing significant potential for industrial and biotechnological applications due to the physicochemical properties of the pigment (Guo et al. 2023).

#### Relevance to biodeterioration

Importantly, DHN-melanin is also the main pigment implicated in black mold stains and fungal biodeterioration of historical and artistic materials, including paper, parchment, and wood. Tavzes et al. (2009) identified DHN-melanized dematiaceous fungi as the major colonizers of paper and darkened wood-based cultural heritage objects, where their melanin formed chemically stable and recalcitrant deposits that resist both chemical bleaching and microbial biodegradation, an observation that highlights the remarkable durability and environmental persistence of this melanin type (Tavzes et al. 2009).

### Tricyclazole as a specific inhibitory tool

- Mechanism of Action: Recognized as the “gold standard” for DHN-melanin inhibition, tricyclazole (5-methyl-1,2,4-triazol[3,4-b]benzothiazole) specifically targets hydroxynaphthalene reductases (1,3,6,8-THN and 1,3,8-THN reductases). This action prevents the conversion of 1,3,6,8-THN to scytalone and 1,3,8-THN to vermelone without inhibiting fungal viability (Bell and Wheeler 1986; Eisenman and Casadevall 2012b; Chhoker et al. 2025).
- Accumulation of Metabolites: This inhibition results in the accumulation of soluble, colored shunt products, such as flaviolin (derived from the oxidation of accumulated 1,3,6,8-THN) and 2-hydroxyjuglone (2-HJ), allowing for the visual and chemical identification of the pathway blockade (Bell and Wheeler 1986; Franzen et al. 2006).
- Experimental Application and Results: Treatment with tricyclazole generates “albino” or pigment-deficient phenotypes. In *F. pedrosoi*, the use of 16 µg/mL of tricyclazole resulted in non-pigmented conidia and sclerotic cells, which although viable, exhibited alterations in the cell wall surface and became significantly more susceptible to killing by neutrophils and oxidants (Franzen et al. 2006; Cunha et al. 2010). In phytopathogens such as *P. oryzae*, tricyclazole prevents disease (rice blast) by impeding plant penetration, thereby serving as the basis for anti-penetrating fungicides (Bell and Wheeler 1986).

### L-DOPA melanin (Eumelanin)

L-DOPA melanin, also known as eumelanin, is a dark, nitrogen-containing pigment widely distributed across biological kingdoms. It represents the primary melanin pigment in animals (Bell and Wheeler 1986; Simon and Peles 2010; Guo et al. 2023; Koehler et al. 2024) and is also synthetized by various bacteria (Singh et al. 2021; Muñoz-Torres et al. 2024). Several pathogenic fungi, such as *Basidiomycetes*, most notably *Cryptococcus* sp. (Eisenman and Casadevall 2012b; Nosanchuk et al. 2015; Chhoker et al. 2025); and *Ascomycetes*, including *Histoplasma capsulatum* (Mihu and Nosanchuk 2012), *Paracoccidioides* sp. (Gómez et al. 2001; Emidio et al. 2020) and *Candida* sp. (Morris-Jones et al. 2005; Almeida-Paes et al. 2021) are able to synthesize DOPA-melanin, often referred to as L-DOPA melanin because of its biosynthetic precursor, L-3,4-dihydroxyphenylalanine. Dematiaceous fungi, such as *F. pedrosoi* can also produce this type of melanin in addition to DHN-melanin, with the relative levels of each melanin dependent on growth condition and substrate availability (Koehler et al. 2024).

#### Biosynthesis of L-DOPA melanin

The L-DOPA melanin is synthesized from the amino acid L-tyrosine (Bell and Wheeler 1986; Simon and Peles 2010; Pralea et al. 2019; Muñoz-Torres et al. 2024). The enzyme tyrosinase catalyzes the hydroxylation of L-tyrosine to DOPA, and subsequently oxidizes DOPA to dopaquinone (Bell and Wheeler 1986; Simon and Peles 2010; Pralea et al. 2019; Singh et al. 2021; Muñoz-Torres et al. 2024). In *C. neoformans*, oxidation of DOPA to dopaquinone is catalyzed by a cell wall-associated laccase, a key enzyme in the melanization pathway (Eisenman and Casadevall 2012b). The resulting dopaquinone, as a highly reactive intermediate, undergoes spontaneous intramolecular cyclization to form dopachrome (Simon and Peles 2010; Pralea et al. 2019; Singh et al. 2021). Dopachrome is then either spontaneously or enzymatically converted by a Tyrosinase-Related Protein 2 (Tyrp2), into dihydroxyindoles, primarily 5,6-dihydroxyindole (DHI) and 5,6-dihydroxyindole-2-carboxylic acid (DHICA) (Borges et al. 2001; Simon and Peles 2010; Pralea et al. 2019; Guo et al. 2023; Wakamatsu and Ito 2023; Muñoz-Torres et al. 2024). These indole units undergo additional oxidative polymerization and cross-linking to form the final eumelanin complex polymer (Bell and Wheeler 1986; Simon and Peles 2010).

The critical role of laccase in L-DOPA melanin formation has been demonstrated in several fungal models. *C. neoformans* express two laccases, Lac1 and Lac2, which are localized to the cell wall and cytoplasm, respectively. In *C. neoformans*, Lac1-/- mutants fail to synthesize melanin even in the presence of L-DOPA, resulting in albino phenotypes with attenuated virulence (Zhu and Williamson 2004). Similarly, laccase-deficient mutants in *Fusarium oxysporum* show a complete loss of pigmentation, reduced stress tolerance, and impaired colonization capacity (Cañero and Roncero 2008). These findings confirm that laccase activity is indispensable for eumelanin biosynthesis, and underscore its potential as a target for antifungal drug development.

#### Distribution of L-DOPA melanin and biological roles

L-DOPA melanin plays a multifaceted role in fungal survival and virulence. In melanized fungi, L-DOPA melanin is typically observed at the cell surface, where it integrates with polysaccharides, lipids, and proteins, forming a composite matrix that enhances rigidity and impermeability (Eisenman and Casadevall 2012b). Specifically in *C. neoformans*, melanization occurs predominantly through the laccase (Lac1) oxidation of exogenous catecholamine substrates, such as DOPA, norepinephrine and dopamine. The resulting melanin is subsequently deposited between the plasma membrane and the cell wall, forming a concentric, electron-dense layer observable by transmission electron microscopy (Eisenman et al. 2007; Nosanchuk et al. 2015). Notably, extracellular vesicles (EVs) secreted by *C. neoformans* contain laccase, and are associated with the incorporation of L-DOPA melanin into these membranous structures, and pigment distribution to the cell surface or the extracellular surrounding environment (Rodrigues et al. 2008; Eisenman et al. 2009). In *C. neoformans*, melanin confers resistance against oxidative and nitrosative stress, UV and gamma radiation, and antifungal compounds, while also enhancing survival within macrophages and attenuating host immune responses (Eisenman and Casadevall 2012b; Nosanchuk et al. 2015). Melanin-deficient mutants are markedly less virulent, highlighting its essential role in cryptococcal pathogenesis. The role of L-DOPA melanin has also been described in pathogenic *Ascomycetes*. *H. capsulatum* produces DOPA-derived melanin, which localizes to the cell wall and extracellular vesicles (EVs) and contributes to resistance against amphotericin B and oxidative damage (van Duin et al. 2002; Nosanchuk et al. 2015). Similarly, recent studies (Smith et al. 2022) revealed melanin-like pigments in the emerging thermotolerant yeast *C. auris*, particularly under oxidative and nutrient-limiting conditions, where melanization was linked to enhanced cell wall rigidity, antifungal tolerance, and macrophage survival suggesting that eumelanin-like polymers may contribute to its environmental resilience and persistence in hospital settings. Overall, this structural arrangement and the dual localization of L-DOPA melanin represent a convergent adaptation across diverse organisms, providing structural/mechanical reinforcement, environmental resilience, stress tolerance, and pathogenic advantages, such as immune evasion and antifungal resistance.

### Pheomelanin

Pheomelanin is a yellow to-reddish-brown sulfur-containing pigment, primarily found in mammals, and responsible for the coloration of red hair and freckles (Simon and Peles 2010; Pralea et al. 2019; Guo et al. 2023). It is distinct from eumelanin in both composition and photochemical behavior, and as their synthesis pathway diverges from eumelanin at the dopaquinone step, it is mainly composed of benzothiazine and benzothiazole units derived from sulfur-containing precursors (Simon and Peles 2010; Pralea et al. 2019; Chhoker et al. 2025). Compared to eumelanin, pheomelanin exhibits lower photostability, higher pro-oxidant potential, and increased reactivity to UV-induced damage, characteristics that influence its biological roles and toxicological implications (Simon and Peles 2010; Pralea et al. 2019; Wakamatsu and Ito 2023).

#### Biosynthesis of pheomelanin

The biosynthetic pathway of pheomelanin diverges from that of eumelanin at the dopaquinone step of the L-tyrosine oxidation cascade. Specifically, in the presence of thiol compounds, such as cysteine or glutathione, dopaquinone undergoes a nucleophilic addition to form cysteinyldopas, predominantly 5-*S*-cysteinyldopa and 2-*S*-cysteinyldopa (Borges et al. 2001; Simon and Peles 2010; Pralea et al. 2019; Li et al. 2021; Guo et al. 2023; Wakamatsu and Ito 2023). These sulfur-containing intermediates are then oxidized to yield benzothiazine and benzothiazole derivatives, which then undergo spontaneous polymerizations into pheomelanin macromolecules (Borges et al. 2001; Simon and Peles 2010; Pralea et al. 2019; Guo et al. 2023; Wakamatsu and Ito 2023). In contrast to eumelanin, pheomelanin biosynthesis requires cysteine availability and typically occurs under conditions of low tyrosinase activity or oxidative stress, favoring sulfur incorporation over indolic ring formation (Simon and Peles 2010; Li et al. 2021).

#### Distribution of pheomelanin and biological roles

Although pheomelanin is the dominant pigment in animals, its occurrence in microorganisms, especially fungi, has been less commonly reported. One of the best documented example is the *Basidiomycete Auricularia auricula-judae*, which is noted to produce a sulfur-rich pheomelanin contributing to its brownish-red pigmentation (Liu et al. 2019; Li et al. 2021). In this species, freezing alters the balance between pheomelanin and eumelanin synthesis, resulting in a lighter color. This change in color is associated with downregulation of proteins involved in tyrosine metabolism and arginine biosynthesis, while antioxidant-related proteins such as peroxidases and superoxide dismutases are upregulated (Li et al. 2021). A pheomelanin-like pigment with a free radical scavenging activity has also been isolated and identified from the sporocarp and mycelium of the saprophytic fungi *Lachnum singerianum* (Ye et al. 2011).

Despite isolated reports, the existence of authentic pheomelanin in fungi remains controversial (Guo et al. 2023). Several studies suggest that yellowish pigments identified as pheomelanin with sulfur detected by elemental analysis may instead represent oxidized variants of eumelanin or mixed melanin polymers containing sulfur traces from cellular thiols (Ye et al. 2011). Therefore, analytical challenges arise because spectroscopic and chemical degradation profiles of mixed melanins frequently overlap, making the definitive structural attribution difficult. Additionally, the absence of canonical cysteinyldopa pathway enzymes in most fungal genomes argues that true pheomelanin formation in fungi is rare and may occur only under exceptional physiological or environmental conditions, such as sulfur enrichment or altered redox balance (Gessler et al. 2014).

### Pyomelanin

Pyomelanin is a typically yellowish-brown, extracellular, and water-soluble melanin produced through the L-tyrosine catabolic pathway (Singh et al. 2021; Guo et al. 2023; Chhoker et al. 2025). It is synthesized by many bacteria and by a more restricted group of fungi (Singh et al. 2021; Muñoz-Torres et al. 2024). Pyomelanin is secreted to the extracellular environment rather than deposited on the fungal cell wall, as for eumelanin and DHN-melanin.

#### Biosynthesis of pyomelanin

The pyomelanin monomeric precursor originates from the catabolism of L-tyrosine and L-phenylalanine rather than a dedicated polymerization pathway. In this pathway, L-tyrosine is first deaminated to *p*-hydroxyphenyl pyruvate, which is then oxidized by 4-hydroxyphenylpyruvate dioxygenase (HppD), in which through a series of oxidation/decarboxylation, is converted to the precursor homogentisic acid (Singh et al. 2021; Guo et al. 2023; Chhoker et al. 2025). Under cellular homeostasis, homogentisic acid (HGA) is cleaved by homogentisate 1,2-dioxygenase (HmgA), which prevents pigment formation. However, when this enzyme is absent or inhibited, HGA accumulates and is secreted from the cell. In the extracellular space, HGA auto-oxidizes into benzoquinone acetic acid, which subsequently polymerizes into the characteristic pyomelanin pigment (Chhoker et al. 2025). Since the pyomelanin formation is tightly linked to tyrosine catabolism, its production is often stimulated by conditions that increase tyrosine turnover or impair HmgA function (Schmaler-Ripcke et al. 2009; Almeida-Paes et al. 2018; Singh et al. 2021).

#### Distribution of pyomelanin and biological roles

Pyomelanin has been described in several bacteria, including *Pseudomonas* sp. (Yabuuchi and Ohyama 1972; Kurian et al. 2014), and *Legionella pneumophila* (Steinert et al. 2001; Chatfield and Cianciotto 2007). This pigment has been also reliably detected in several fungal species, including *Aspergillus* spp. (Schmaler-Ripcke et al. 2009; Palonen et al. 2017; Perez-Cuesta et al. 2020; Koch et al. 2023), C. *neoformans* (Frases et al. 2007), thermodimorphic (Almeida-Paes et al. 2018) and dematiaceous fungi (Fernandes et al. 2021).

In fungi, the pyomelanin pigment is synthesized extracellularly, as evidenced by the detection of HGA in culture supernatants. Since this pigment is water-soluble, it can diffuse into the cellular environment, but it can also deposit and loosely associate with the outer cell surface (Schmaler-Ripcke et al. 2009). However, the extracellular distribution of this pigment is strictly related to its biological activity. The presence of pyomelanin has been correlated to stress tolerance (DeAraujo et al. 2015), such as protection against oxidative stress by scavenging ROS (Schmaler-Ripcke et al. 2009; Li et al. 2021) and metal binding, particularly iron, which may support survival in iron-limited niches (Zheng et al. 2013; Li et al. 2021). These redox activity, metal-binding ability, and water-solubility biochemical features warrant important biotechnological applications of pyomelanins, including bioremediation, antioxidant and radical-scavenging formulations, and biomaterial coatings, due to their ability to form stable, amorphous polymers (Turick et al. 2010; Lorquin et al. 2022). Unlike eumelanin and DHN-melanin, pyomelanin plays a more metabolic and extracellular role in fungal biology, with ongoing research needed to fully elucidate its contribution to fungal virulence.

### Other types of melanins

Although DHN-melanin, L-DOPA melanin, pheomelanin, and pyomelanin represent the major categories of biological melanins, several less common types of melanin have been reported (Solano 2014). These additional melanins are typically substrate-dependent and often originate from unique metabolic intermediates found in specific organisms. Their structures remain less well characterized, but historical and modern analyses confirm their existence in select fungal groups.

#### Neuromelanin

Neuromelanin is a complex mixed-type melanin predominantly found in human catecholaminergic neurons, particularly in the substantia nigra and locus coeruleus (Zecca et al. 2001; Simon and Peles 2010; Haining and Achat-Mendes 2017; Pralea et al. 2019; Guo et al. 2023; Wakamatsu and Ito 2023; Chhoker et al. 2025). Although absent in microorganisms, this pigment is often referred to as a hybrid structural composition, including a pheomelanin-like sulfur-containing core surrounded by a eumelanin-like indole-based surface (Simon and Peles 2010; Pralea et al. 2019; Cao et al. 2021; Guo et al. 2023).

#### GDHB-melanin and catechol-melanin

Distinct from the classic DHN and DOPA pathways, certain *Basidiomycotina* utilize specific phenolic precursors for melanization. As noted by Bell and Wheeler (1986), these melanins are synthesized from γ-glutaminyl-4-hydroxybenzene (GHB) and catechol, respectively. Specifically, in the button mushroom *Agaricus bisporus*, the synthesis of GHB-melanin is a critical physiological process associated with both spore wall formation and the enzymatic browning of fruit bodies (Bell and Wheeler 1986). The pathway involves the hydroxylation of GHB to γ-glutaminyl-3,4-dihydroxybenzene (GDHB) by tyrosinase/polyphenol oxidases, followed by oxidation to quinones that polymerize into the final pigment. While GHB-melanin is predominant in *A. bisporus* spores, catechol-melanin results from the direct oxidation of catechol and has been utilized in biosorption studies for structural characterization. Recent reviews highlight that understanding these alternative pathways is essential for metabolic engineering applications, particularly in manipulating the browning phenotype in commercial mushroom strains (Bell and Wheeler 1986; Weijn et al. 2013; Qin and Xia 2024; Chhoker et al. 2025).

### Molecular characteristics and resistance to degradation

Regardless of their precursor and biosynthetic origins, all melanins share molecular and physicochemical properties that confer exceptional stability and resistance to degradation. Melanins are amorphous, heterogeneous, and notoriously insoluble in most aqueous and organic solvents (Bell and Wheeler 1986; Simon and Peles 2010; Nosanchuk et al. 2015; Pralea et al. 2019; Guo et al. 2023; Wakamatsu and Ito 2023; Muñoz-Torres et al. 2024; Chhoker et al. 2025). They are resistant to concentrated acids and only partially soluble in strong alkali, a property used experimentally for dissolution and extraction, though this process induces structural alterations and fragmentation (Pralea et al. 2019; Singh et al. 2021; Guo et al. 2023; Chhoker et al. 2025). At the molecular level, melanins are built from highly conjugated aromatic units linked through non-hydrolysable carbon–carbon and carbon–nitrogen bonds, generating a disordered yet tightly cross-linked macromolecular network (Wakamatsu and Ito 2023). Ultrastructural studies reveal melanin occurs as aggregated spherical granules, estimated to be 40–200 nm in diameter (Eisenman and Casadevall 2012b; Nosanchuk et al. 2015; Cordero and Casadevall 2017; Pralea et al. 2019; Chhoker et al. 2025). These granules are synthesized within melanosomes or melanosome-like vesicles, intracellular compartments dedicated to pigment synthesis and assembly (Walker et al. 2010; Eisenman and Casadevall 2012b; Chhoker et al. 2025). Following their synthesis, melanin granules are transported to and incorporated into the cell wall, where they become covalently anchored to chitin and other polysaccharides, strengthening the rigidity and limiting permeability of this structure (Zhong et al. 2008; Eisenman and Casadevall 2012b; Nosanchuk et al. 2015; Cordero and Casadevall 2017; Chhoker et al. 2025). X-ray diffraction and spectroscopic analyses indicate that melanin granules consist of stacked, planar aromatic sheets, arranged in a graphite-like, partially ordered architecture (Nosanchuk et al. 2015; Kim et al. 2016a; Cordero and Casadevall 2017; Chhoker et al. 2025). This dense, cross-linked, and aggregated structure creates a polymer that is profoundly resistant to chemical degradation, including oxidants and acids (Wakamatsu and Ito 2023), enzymatic digestion due to steric hindrance of internal targets (Bell and Wheeler 1986; Eisenman and Casadevall 2012a) and high temperatures resistance up to 600 °C (Guo et al. 2023; Chhoker et al. 2025). Altogether, these molecular and ultrastructural features make melanin one of the most recalcitrant biological polymers, posing a significant challenge for degradation-based approaches and motivating ongoing research into innovative physical, chemical, and biological strategies to overcome melanin’s intrinsic resistance.

Also structural recalcitrance in fungal melanins is significantly shaped by environmental origin, with hypersaline habitats inducing unique architectural adaptations. The halotolerant black yeast *Hortaea werneckii* exemplifies this by incorporating DHN-melanin into its cell wall to minimize glycerol loss under osmotic stress. In marine niches, melanin granule organization is salt-dependent, forming a dense protective barrier essential for cell wall integrity and volume-recovery capacity (Kejžar et al. 2013; Rani et al. 2013). As opposed to terrestrial fungal melanins, which often serve as primary antioxidants, evidence suggests that defense against H_2_O_2_ is primarily mediated by intracellular systems rather than the pigment itself. Nevertheless, these marine-derived melanins exhibit distinct biological potential, including possible antibacterial activity, suggesting that their complex architectural density may harbor novel functional properties compared to terrestrial models (Rani et al. 2013).

## Mechanisms and strategies for melanin degradation

### Physical methods

**Fig. 2:**
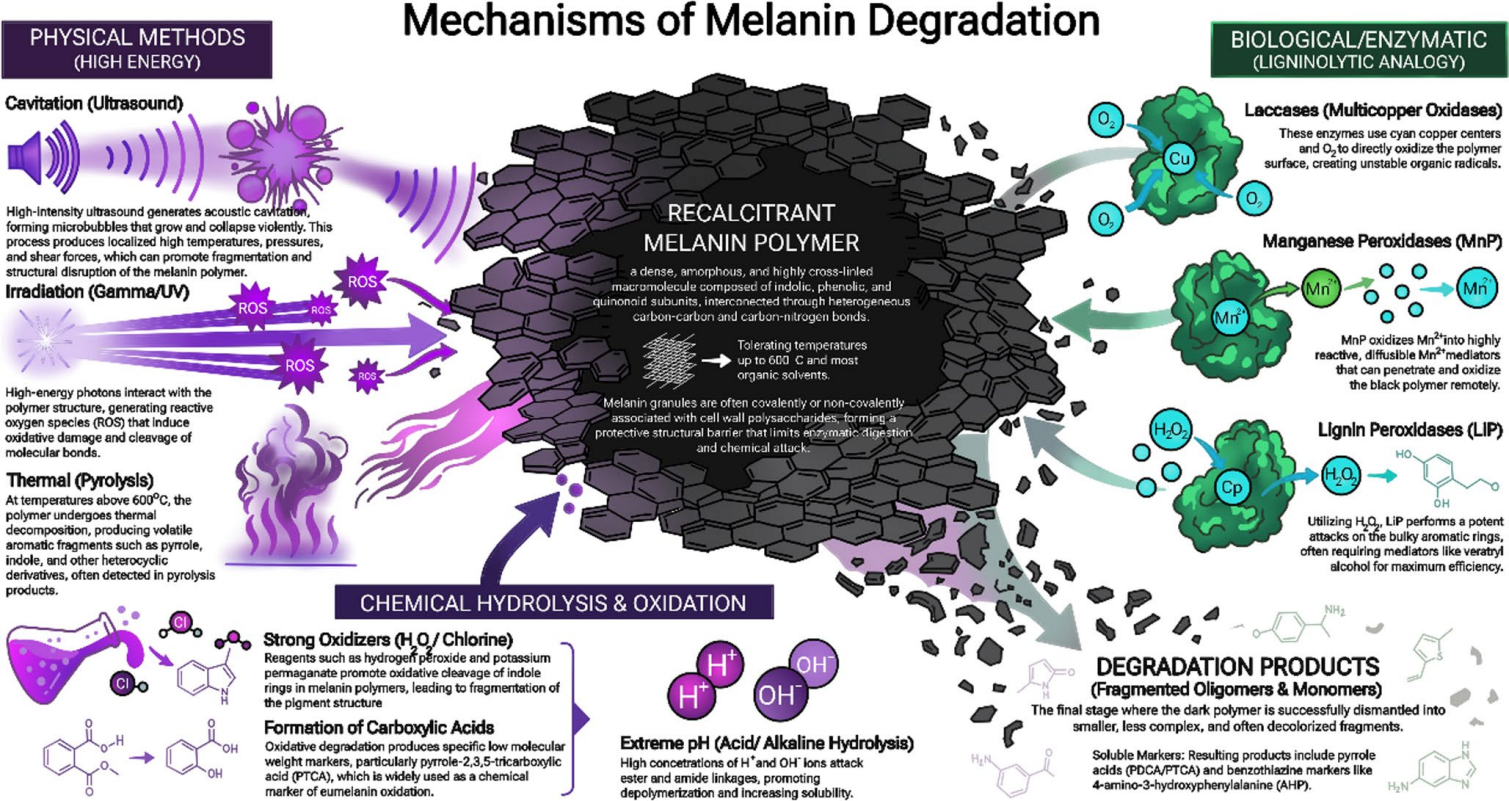
Mechanisms of melanin degradation. This visual summary outlines the physical, chemical, and biological approaches discussed in the review for dismantling the recalcitrant polymer melanin. It highlights the connections between these technical strategies, practical applications, and the resulting breakdown products

Physical methods for melanin degradation remain the least explored among available approaches, primarily because melanin is known for its extraordinary physicochemical stability and resistance to thermal, photochemical, and mechanical disruption (Eisenman and Casadevall 2012a; Cordero and Casadevall 2017). Nevertheless, certain physical forces can induce partial degradation, fragmentation, or structural weakening of melanin polymers (Fig. 2).

#### Photodegradation

Melanin’s fundamental biological role is photoprotection, achieved through its stability to absorb and safely dissipate UV and visible radiation (Zonios et al. 2008; Cordero and Casadevall 2017; Guo et al. 2023). Melanin displays a broad and monotonic absorption spectrum, high in the UV range, that decreases exponentially toward the visible region (Zonios et al. 2008; Pralea et al. 2019; Guo et al. 2023). However, the photodegradation behavior differs depending on the melanin type. Eumelanin is strongly photoprotective, characterized by an ultrafast non-radiative decay that dissipates the absorbed energy as heat within picoseconds (Simon and Peles 2010; Cordero and Casadevall 2017). This efficient internal energy conversion makes eumelanin extremely resistant to photo-oxidative breakdown. In contrast, pheomelanin exhibits phototoxicity, demonstrating a lower photoionization threshold (~ 326 nm), significantly yielding reactive oxygen species (ROS), such as superoxide radicals (Panzella et al. 2010), upon UV-A exposure (Tanaka et al. 2018), leading to its own degradation and simultaneously damaging DNA and cellular biomolecules (Simon and Peles 2010). In turn, mixed or hybrid melanins may exhibit intermediate photostability depending on their sulfur content, aromatic density, and metal coordination states (d’Ischia et al. 2015; Solano 2016). Overall, photodegradation may fragment or alter the melanin oxidation state, depending on its specific resistance capacity to photolysis; therefore, it is not considered an efficient strategy for removing established melanin deposits, particularly DHN-melanin or eumelanin (Ito et al. 2018; Mattoon et al. 2021; Upadhyay et al. 2022).

#### Thermal degradation

Melanin is one of the most thermally resistant biopolymers (Singh et al. 2021; Suthar et al. 2023). Thermogravimetric analysis of natural and synthetic melanins shows only minor mass loss at low temperatures (~ 200–250 °C), which corresponds to the evaporation of bound water and partial CO_2_ release at 350 °C, while the bulk remains intact (Singh et al. 2021; Suthar et al. 2023). At temperatures above 500 °C, eumelanin, pheomelanin, and DHN-melanin begin to undergo significant pyrolysis, producing several aromatic compounds, such as benzene, pyrrole, indole and their derivatives (Guo et al. 2023; Chhoker et al. 2025). From a practical standpoint, due to this remarkable stability, thermal degradation is not a feasible strategy for eliminating melanin deposits in vivo or for cleaning melanin-based stains from cultural-heritage materials. The remarkable preservation of melanin signatures in thermally altered and fossilized tissues over geological timescales further underscores its resistance to thermal and chemical degradation (Al-Shamery et al. 2025). Consequently, high-temperature treatment of melanin, rather than being an effective approach for targeted depigmentation, is confined to applications in analytical and materials-science (Fig. 2). For example, since the pyrolytic process generates new compounds, it is often coupled to gas chromatography–mass spectrometry (Py-GC-MS), and therefore used for melanin structural profiling (Pralea et al. 2019).

#### Laser irradiation

Laser-induced disruption of melanin is widely used in dermatology to treat hyperpigmentation. Conceptually, short pulses of light that match the absorption spectrum of the target chromophore are converted into heat faster than it can diffuse away, allowing preferential heating of pigmented structures, such as melanosomes within keratinocytes and melanocytes (Anderson and Parrish 1983; Watanabe 2008). Histologic and ultrastructural studies demonstrate selective photo thermolysis and rupture of melanosomes, while preserving surrounding tissue (Brazzini et al. 2001; Kim et al. 2012). From the perspective of melanin degradation, laser irradiation can indeed fragment melanin granules and alter their oxidation state. However, the high localization of laser energy, equipment specifications/ set up and substrate-specific performance account for melanin degradation and clinical applications (Garg et al. 2024). Regarding microbial melanin, it is typically embedded in a thick cell wall and associated with other biopolymers, which impair heterogeneous absorption, making the effective disruption of microbial melanin by laser technically impractical (Eisenman and Casadevall 2012a; Camacho et al. 2019; Qin and Xia 2024). Therefore, although laser irradiation provides a powerful proof-of-concept that melanin can be disrupted, its application to microbial or environmental melanin degradation remains extremely limited, but suited to clinical and conservation contexts (Tserevelakis et al. 2020; Sanmartín et al. 2021; Heppner et al. 2024).

#### Ultrasound-assisted melanin extraction/degradation

High-intensity ultrasound is widely used as an auxiliary physical method for melanin extraction, demonstrating how mechanical energy can disrupt the melanin matrix without fully destroying it (Zou et al. 2010; Suthar et al. 2023). Ultrasound drives the formation, growth and implosive collapse of microbubbles in a liquid (Fig. 2). The collapse of these bubbles near solid surfaces or microbial cell walls results in acoustic cavitation (Lu et al. 2020). In the context of microbial melanin, these cavitation-driven effects are primarily used to extract rather than degrade melanin. Ultrasound-assisted extraction (UAE) has become a standard method for recovering melanin from various fungi, yielding higher yields and shorter extraction times than conventional alkali–acid protocols (Singh et al. 2021; Suthar et al. 2023). Seminal studies conducted on *Auricularia auricula* demonstrated that probe-type ultrasound (250 W), combined with alkali (1 M NaOH), significantly improved melanin extraction while preserving its spectral and redox properties (Zou et al. 2010, 2015). Similar UAE strategies were applied to extract melanin from solid-state fermentation substrates of *Inonotus hispidus* and other *Basidiomycetes*. Extending UAE times and cycles facilitated disruption of cellular structures and melanin dissolution; however, excessive temperatures and ultrasonication duration may compromise the stability and quality of the extracted melanin due to oxidation (Hou et al. 2019). For the phytopathogen *Sporisorium reilianum*, acid hydrolysis (4 h at 60 °C), followed by ultrasonic-assisted alkali-solubilization (pH 13, 240 W, 2 h at 50 °C) and acid precipitation/centrifugation yielded the best results without fully destroying the pigment’s function (Lu et al. 2020). Mechanistically, UAE cavitation improves solvent penetration into fungal cell walls, disrupting the polysaccharide–protein network, weakening pigment–matrix interactions, and promoting detachment of melanin granules that are covalently or noncovalently anchored to wall components (Eisenman and Casadevall 2012b; Suthar et al. 2023).

#### Microwave-assisted extraction (MWE)

Similarly to the UAE, microwave irradiation consists of another physical process for perturbing melanized structures, mainly exploited as a pre-treatment method for extraction rather than pigment degradation (Singh et al. 2021; Guo et al. 2023). In aqueous media, irradiated microwaves on polar molecules generate rapid dielectric heating and expansion, which can fracture and perforate cell walls and enhance membrane permeability (Lozano Pérez et al. 2024). In biomass systems, this may lead to cell disruption and damage to organelles, facilitating the dispersion of solid matrix components to the surrounding solvent. For fungal melanin, several microwave-assisted extraction (MAE) workflows have been proposed. Lu et al. (2014) proposed an MAE protocol for extracting of intracellular melanin from the saprophytic fungi *Lachnum singerianum* YM296 (LIM), to evaluate its antiradiation, antioxidant, and anti-aging activities (Lu et al. 2014). The optimized conditions consisted of 1.05 mol/L NaOH solution concentration, a solid material–liquid ratio of 1:14.72 (g/mL), and a microwave power of 320 W operated for 118.70 s. This protocol resulted in an 11.08% LIM extraction yield, which was 40.43% higher than yields from traditional thermal or conventional solvent protocols, while preserving the pigment’s key structural and redox properties. Later, Ma et al. (2023) optimized a MAE protocol for extracting natural melanin from *Auricularia heimuer* fermentation, demonstrating that microwave time and power (300 W for 50 min), in combination with alkali-solubilization (3 M NaOH, pH 12, for 2 h at 70 °C) and acid precipitation (1.0 M HCl, pH 3.0, for 3 h at 70 °C), significantly increased pigment extraction yield and retained all the typical features of melanin, such as a monotonic UV–visible spectrum, characteristic Fourier transform infrared (FT-IR) spectroscopy bands, granular morphology by scanning electron microscopy (SEM) (Ma et al. 2023). Taken together, these techniques represent controlled physical extraction methods (Singh et al. 2021), functioning as green, intensification tools that make subsequent chemical or biological degradation more efficient rather than providing an independent solution for removing established melanin deposits.

#### Mechanical fragmentation

Mechanical fragmentation encompasses techniques such as high-pressure homogenization, bead beating, French press disruption, and various milling approaches, all relying on intense shear, cavitation, and impact to the rupture of microorganism cell walls. In high-pressure homogenization, cell suspensions are forced through a narrow orifice, causing rapid deformation and failure of the wall–membrane system (Nemer et al. 2021). Instead, bead beaters rely on collisions between cells and grinding matrix, such as glass, zirconia or steel beads, under vigorous agitation (Klimek-Ochab et al. 2011). For the extraction of intracellular or cell-wall-associated pigments, combinations of mechanical cell-disruption technologies can significantly increase the pigment extraction yields compared to using either physical or chemical methods alone (Nemer et al. 2021). Regarding melanin extraction, these treatments would mainly lead to the fragmentation of cell walls and biomolecular matrix, in which the pigment is embedded, rather than damaging the melanin polymer backbone, thereby releasing structurally and functionally intact melanin granules. In the context of melanin degradation, therefore, mechanical methods should be viewed as enabling technologies that facilitate downstream chemical or enzymatic attack, rather than as independent strategies capable of dismantling the melanin polymer.

### Chemical methods

Most chemical methods used for melanin degradation rely on strong or reducing agents. Due to their harsh nature and lack of selectivity, these approaches are generally unsuitable for routine cosmetic or industrial applications and are primarily employed for the analytical characterization of melanin structure or highly controlled depigmentation of melanin-containing tissues and materials (Pralea et al. 2019; Guo et al. 2023; Wakamatsu and Ito 2023).

#### Analytical degradation

The primary purpose of analytical degradation is not the complete destruction of the pigment, but rather the controlled cleavage of the recalcitrant polymer into specific, low molecular-weight monomeric markers used for structural elucidation and quantification (Pralea et al. 2019). Due to the high insolubility of melanin in water and most organic solvents, sodium hydroxide (NaOH) plays a pivotal role in these protocols, serving both as a solubilizing agent for physicochemical characterization (Noman et al. 2022) and as the alkaline medium required to ensure the complete degradation of biological matrices, such as hair, during oxidative procedures (Borges et al. 2001). This controlled chemical breakdown, often achieved through alkaline hydrogen peroxide oxidation or acidic hydrolysis, allows for the precise identification of eumelanin and pheomelanin contributions within complex heterogeneous structures (Ito and Wakamatsu 1998; Ito et al. 2011; Wakamatsu and Ito 2023).

#### Eumelanin (DHI/DHICA) degradation

For the chemical degradation of eumelanin, one of the most widely applied strategies is the alkaline hydrogen peroxide (H_2_O_2_) oxidation, typically carried out in carbonate or borate buffer at high pH (Borges et al. 2001; Pralea et al. 2019; Singh et al. 2021; Guo et al. 2023; Wakamatsu and Ito 2023). Under alkaline conditions, the generated hydroperoxide ions (OOH⁻) perform a nucleophilic attack on the indole ring units (DHI and DHICA) of eumelanin, leading to a ring-opening reaction of the indole units (Singh et al. 2021; Guo et al. 2023). The main degradation products are pyrrole-2,3-dicarboxylic acid (PDCA) and pyrrole-2,3,5-tricarboxylic acid (PTCA), which serve as specific markers for DHI- and DHICA-derived units, respectively (Borges et al. 2001; Wakamatsu and Ito 2002; Simon and Peles 2010). Additional ring-opened pyrrole acids such as pyrrole-2,3,4-tricarboxylic acid (isoPTCA) and pyrrole-2,3,4,5-tetracarboxylic acid (PTeCA) can be formed from more highly oxidized or cross-linked eumelanin structures, as identified in fossil and tissue melanin studies (Glass et al. 2012; Pinheiro et al. 2019). The chemical degradation of eumelanin is a critical analytical strategy for quantifying its monomeric composition and structural units. Alkaline hydrogen peroxide (H₂O₂) oxidation is a widely used method that degrades eumelanin into specific markers: pyrrole-2,3-dicarboxylic acid (PDCA), which is derived from 5,6-dihydroxyindole (DHI) units, and pyrrole-2,3,5-tricarboxylic acid (PTCA), derived from 5,6-dihydroxyindole-2-carboxylic acid (DHICA) units (Borges et al. 2001; Wakamatsu and Ito 2002). Borges et al. (2001) reported reproducible yields of 0.37% for PDCA and 4.8% for PTCA when human hair eumelanins were subjected to this method, demonstrating its utility for estimating the ratio of DHI to DHICA units in biological samples (Borges et al. 2001).

A classical alternative and standard analytical method is acidic potassium permanganate (KMnO_4_) oxidation (Pralea et al. 2019; Guo et al. 2023) (Fig. 2). This protocol preferentially oxidizes DHICA-derived structures, yielding PTCA as a highly specific marker for DHICA-melanin, with yields around 2.8% for synthetic DHICA-melanin but negligible amounts of PDCA (Wakamatsu and Ito 2002). While highly specific, the KMnO_4_ method underestimates DHI-derived units (Wakamatsu and Ito 2002). In contrast, the alkaline H₂O₂ oxidation method is simpler and provides a higher yield of PDCA (approximately 10-fold higher than acidic KMnO_4_), making it superior for characterizing DHI-rich eumelanins like dopamine-melanin (Wakamatsu and Ito 2023). Collectively, these oxidative protocols form the foundation of modern high-performance liquid chromatography (HPLC) methods for quantifying eumelanin in diverse biological materials, including hair, skin, and cultured melanocytes (Ito et al. 2011; Pralea et al. 2019). Specifically, the ratio of PDCA to PTCA obtained via alkaline H₂O₂ oxidation serves as a reliable indicator of the DHI/DHICA ratio, allowing for the chemical phenotyping of pigmentation (Ito et al. 2011).

#### Pheomelanin (Cysteinyldopa) degradation

The degradation of pheomelanin can be achieved through reductive hydrolysis using hot hydriodic acid (HI) (Borges et al. 2001; Pralea et al. 2019; Wakamatsu and Ito 2023). This process specifically cleaves the sulfur-containing benzothiazine and benzothiazole pheomelanin units to release their characteristic markers: 4-amino-3-hydroxyphenylalanine (AHP), derived from 5-S-CysDopa and 3-aminotyrosine (3AT), derived from 2-*S*-CysDopa hydrolysis (Borges et al. 2001; Simon and Peles 2010; Wakamatsu and Ito 2023). In the same study conducted on eumelanin, Borges et al. (2001) reported relatively high yields of 23% for AHP and 16% for 3AT, indicating a high efficiency and specificity of this reductive approach (Borges et al. 2001). Complementary oxidative protocols using alkaline H₂O₂ or KMnO₄ also generate thiazole-4,5-dicarboxylic acid (TDCA) and thiazole-2,4,5-tricarboxylic acid (TTCA) as markers of benzothiazole-type pheomelanin (Ito et al. 2011; Pralea et al. 2019; Guo et al. 2023).

#### Applied degradation (Chemical mediators and bleaching systems)

In applied settings, chemical degradation aims at bleaching or partial breakdown of melanin in tissues, biomass or materials. Many chemical agents commonly used in cosmetics, such as hydroquinone, kojic acid or arbutin, are primarily melanin synthesis inhibitors, acting on tyrosinase or upstream signaling/reactions rather than degrading pre-existing pigments (Simon and Peles 2010; Panich et al. 2011; Shin et al. 2019; Sadaqat et al. 2020; Li et al. 2021).

The aforementioned H_2_O_2_ and KMnO₄ in higher concentrations or prolonged incubations are considered strong oxidizing agents. Together with sodium hypochlorite or related active chlorine donors can effectively decolorize melanin by oxidative ring opening and fragmentation of the conjugated aromatic system (Smith et al. 2017; Guo et al. 2023; Wakamatsu and Ito 2023). In histopathology, KMnO₄ followed by oxalic acid (or warm diluted H_2_O_2_) consisted of a classical bleaching method for melanin granule removal from heavily pigmented sections (Manicam et al. 2014). More recently, hypochlorous acid (HClO), generated from sodium hypochlorite, completely bleached melanin in tumor tissue sections within ~ 20 min at 1% concentration without compromising hematoxylin–eosin or immunohistochemical staining (Wang and Wang 2024). In hair and skin, H₂O₂ bleaching is often combined with metal-catalyzed radical formation, and both oxidizing and reducing agents, respectively, can modulate melanin’s redox state and color (Smith et al. 2017).

Reducing bleaching agents, such as sodium sulfite and sodium dithionite, and largely used in industry. In the leather industry, after enzymatic dehairing and fiber-opening of buff calfskins, 1% sodium sulfite or sodium dithionite achieved complete removal of visible melanin patches, as confirmed by UV- 300 nm peak disappearance and SEM (Punitha et al. 2009). Besides their applications in melanin depigmentation, sodium dithionite and sodium sulfite are also used in the paper and textile industries, where they efficiently reduce conjugated double bonds and carbonyl groups in lignin and several dyes (Punitha et al. 2009; El-Sakhawy et al. 2021).

Ascorbic acid (vitamin C) and its derivatives are extensively used as depigmenting agents and act primarily by scavenging ROS, reducing dopaquinone back to DOPA, and reducing melanin content, inhibiting UVA-mediated catalase inactivation, glutathione (GSH) depletion and NO production through downregulation of eNOS and iNOS expression (Panich et al. 2011; Sanadi and Deshmukh 2020).

Finally, Laccase-Mediator Systems (LMS) bridge chemical and biological oxidative approaches. In these systems, fungal laccases oxidize synthetic aromatic mediators, such as 1-hydroxybenzotriazole (HBT) and 2,2’-azinobis(3-ethylbenzothiazoline-6-sulfonate) (ABTS), into stable, diffusible, and highly potent oxidants (cation radicals) capable of non-specific oxidation of the complex melanin polymer, thereby enhancing the oxidative power of enzymes (Tavzes et al. 2009; Khammuang and Sarnthima 2013).

### Biological methods

Biological methods represent the most extensively studied, effective, and potentially safest strategies for melanin degradation (Eisenman and Casadevall 2012a; Guo et al. 2023). These methods often utilize the ligninolytic enzyme systems of white-rot fungi, which recognize melanin as a lignin-analog polymer (Tavzes et al. 2009; Sadaqat et al. 2020; Rathour et al. 2024).

#### Degradation by ligninolytic enzymes (LiP, MnP and Laccase)

The primary enzymes identified for melanin degradation are lignin peroxidase (LiP), manganese peroxidase (MnP), and laccase (Fig. 2) (Tavzes et al. 2009; Eisenman and Casadevall 2012a; Khammuang and Sarnthima 2013; Kim et al. 2016b; a; Sadaqat et al. 2020). The lignin peroxidases (LiP), notably from *Phanerochaete chrysosporium* (Woo et al. 2004; Sadaqat et al. 2020) and bacteria like *Pseudomonas fluorescence* (Rathour et al. 2024), are potent melanin degraders. The degradation mechanism is indirect, requiring H_2_O_2_ (Sung et al. 2019; Rathour et al. 2024) and often a mediator such as veratryl alcohol (VA) (Sadaqat et al. 2020). LiP uses H_2_O_2_ to oxidize VA into a diffusible veratryl alcohol cation radical (VA^•+^), which then performs a non-specific attack on the melanin polymer (Sadaqat et al. 2020). Sadaqat et al. (2020) achieved a 92% decolorization using this LiP-VA system under optimal conditions (pH 3.0, 40 °C). Analysis by SEM and FTIR confirmed degradation, showing “void spaces” on the granules and structural changes (Sadaqat et al. 2020; Rathour et al. 2024). The manganese peroxidase (MnP) was identified as a “melaninase enzyme” in white-rot fungi (Butler and Day 1998; Kim et al. 2016b) and was found to be the primary enzyme responsible for melanin degradation in *Geotrichum* sp. (Kim et al. 2016b; a). The MnP from this fungus, with a molecular weight of 54.2 kDa, required H_2_O_2_ and MnSO_4_, consistent with the standard MnP mechanism where Mn^2+^ is oxidized to the highly reactive Mn^3+^, which acts as the diffusible oxidant (Kim et al. 2016b; a). The process was enhanced by glucose as a co-substrate and was optimal at acidic pH 4.5 (Kim et al. 2016b; a). Tavzes et al. (2009) also confirmed the presence of MnP in extracts from *P. chrysosporium* and its capacity to oxidize DHN-melanin, as shown by an increase in carbonyl (C = O) bands in FTIR spectra (Tavzes et al. 2009). Laccases (Lac) are multicopper polyphenoloxidases found in plants, fungi and bacteria, able to catalyze the oxidation of several aromatic compound (Piscitelli et al. 2010; Cordero and Casadevall 2017; Shin et al. 2019; Singh et al. 2021). Laccase is widely used in biotechnology for its potential to degrade lignin and, in bioremediation, to degrade other environmental pollutants, generating green products. Industrial applications also include fabric whitening and biofuel production. Crude laccase from *Lentinus polychrous* demonstrated a low but significant ability to decolorize synthetic tyrosine-melanin (22% degradation) without any added mediator (Khammuang and Sarnthima 2013). However, clearly its efficacy is dramatically enhanced by mediators; with the synthetic mediator ABTS, *L. polychrous* laccase achieved 87% decolorization (Khammuang and Sarnthima 2013). Similarly, the laccase-HBT system was highly effective in oxidizing DHN-melanin and bleaching mold stains on paper (Tavzes et al. 2009). Natural laccase mediators, such as vanillin, have also been used, achieving 45% decolorization (Khammuang and Sarnthima 2013). Miao et al. (2017) compared the decolorization efficiency of the three lignolitic enzymes, lignin peroxidase (LiP), manganese peroxidase (MnP) and laccase on pheomelanin-enriched samples from human hair and cutaneous melanoma tissues, and synthetic eumelanin (Miao et al. 2017). LiP showed a potent and the best degree of eumelanin and pheomelanin decolorization (40%-70%), followed by MnP (22–42%) and laccase (9–21%) (Miao et al. 2017).

## Synergistic and cellular systems for melanin degradation

### Synergistic enzyme complexes

The combinations of multifunctional enzymes for the engineering of synergistic enzyme complexes have been proposed to work more effectively for melanin degradation, such as skin whitening and other applications. Shin et al. (2019) engineered a bacterial complex merging *E. coli* laccase (CueO), a dye-decolorizing *Bacillus subtilis* peroxidase (DyP), and the dockerin domain of a *Clostridium cellulovorans* endoglucanase B, whose carbohydrate binding module had been replaced with the melanin binding peptide (MBP), resulting in 64% increase in binding to melanin and enhanced degradation (Shin et al. 2019). The laccase oxidized melanin precursors generate H_2_O_2_ in situ, which was then immediately consumed by the peroxidase (DyP) to degrade the melanin polymer. This optimized complex activity in H_2_O_2_ recycling resulted in a 6.4-fold increase in degradation compared to laccase alone, and has been considered as next generation whitening agent.

### Lysosomal degradation

Melanin degradation occurs naturally in human keratinocytes through the action of lysosomal enzymes (Guo et al. 2023). Melanosomes are disintegrated by lysosomal hydrolases, such as acid phosphatase and cathepsins, which break down the inner protein matrix of the organelle (Guo et al. 2023). Specifically, the lysosomal protease cathepsin V (CTSV) plays a critical role in this process; high expression of CTSV is associated with light-colored skin, whereas its expression is reduced in the basal layer of hyperpigmented skin, leading to melanosome stagnation (Homma et al. 2018). Despite this degradation machinery, recent evidence suggests that after fusion with lysosomes, melanin-containing compartments (termed melanokerasomes) may evolve into storage lysosomes. These compartments exhibit reduced hydrolytic activity and retain undigested melanin cargo to ensure long-term photoprotection, a mechanism analogous to the formation of dysfunctional lysosomes in age-related diseases (Neto et al. 2025). Inspired by the lysosomal capacity for pigment processing, biotechnological strategies have been developed using lysosome-related organelle extracts (LOE) from *Saccharomyces cerevisiae*. Jeon et al. (2021) engineered yeast strains to overproduce the antioxidant enzyme glutathione peroxidase (GPX) to enhance this activity. The study demonstrated that LOE from yeast overexpressing GPX2 significantly increased peroxidase activity and achieved a melanin-decolorizing effect superior to that of control strains, demonstrating effectiveness even in reducing melanin content in artificial skin tissue models (Jeon et al. 2021).

### Microbial degradation and bioconversion

While many microorganisms produce melanin as a protective pigment, the current scientific literature indicates that to date, few microorganisms have been identified with the capacity to use pre-formed melanin as a primary carbon source (Luther and Lipke 1980; Bell and Wheeler 1986). Instead, many microorganisms can use precursors from other sources or obtained from the melanin degradation itself, such as L-tyrosine, homogentisic acid (HGA), or malonyl-CoA as the carbon source for the *synthesis* and recycling of their own melanin. A strain of *A. fumigatus* isolated from composted coffee and garden waste demonstrated the ability to utilize L-DOPA melanin as a sole carbon source. While the degradation process appeared complete in laboratory settings after 50 days, isotopic tracing revealed a bioconversion pathway wherein over 50% of the substrate carbon was re-incorporated to synthesize a new, structurally distinct fungal allomelanin, with the remaining carbon recycled into cell proteins, chitin, lipids, polar metabolites, and CO_2_ (Luther and Lipke 1980). Similarly, the yeast-like fungus *Geotrichum* sp. is known to decolorize L-DOPA melanin found in human hair (Bell and Wheeler 1986; Kim et al. 2016b; a). Recent characterization of a *Geotrichum* sp. isolated from wastewater identified manganese peroxidase (MnP) as the primary enzyme responsible for this ligninolytic and melanolytic activity (Kim et al. 2016a). Extending the search to traditional fermented foods, Kim et al. (2020) reported the isolation of *Pediococcus acidilactici* strain PMC48 from Korean perilla leaf kimchi. This bacterium displayed a direct melanin-degrading (bleaching) effect, evidenced by the formation of clear zones in melanin-containing agar, and simultaneously inhibited new melanin synthesis by suppressing tyrosinase activity via antioxidant effects (Kim et al. 2020). These dual properties suggest PMC48 is a promising candidate for cosmetic applications targeting hypermelanosis (Kim et al. 2020). Besides *Aspergillus* sp. and *Geothrichum* sp., other fungi have been reported as able to biodegrade melanin, such as *Stropharia* and *Sporotrichum* genera found in contaminated areas (Mohorčič et al. 2007; Kim et al. 2016a). These melanin decoloring/decomposing microorganisms, by this mechanism of action, could potentially be of great value to develop therapeutic agents for hypermelanosis or skin lightening applications.

### Biotechnological strategies (Immobilization)

A persistent limitation in enzymatic degradation systems is the stability and reusability of free enzymes, which often suffer from denaturation, loss of activity, or poor recovery after catalytic cycles (Rathour et al. 2024). To overcome this challenge, recent biotechnological approaches have focused on enzyme immobilization onto solid or nanostructured supports, enabling enhanced catalytic performance, prolonged operational lifetime, and facile recovery. In a representative study, LiP from *Pseudomonas fluorescence* was immobilized onto super-paramagnetic nanoparticles, generating two hybrid nanobiocatalysts: graphene oxide-coated (LiP@MFO-GO) and chitosan-coated (LiP@MFO-Chit). Both complexes exhibited significant melanin-degrading activity, achieving 41.6% and 47.3% degradation, respectively. Beyond catalytic efficiency, immobilization results also in greater thermostability, resistance to pH variations, and enabled magnetic separation, allowing enzyme recovery and reuse over multiple degradation cycles with minimal loss of activity (Rathour et al. 2024). In summary, these nanocomposite-based immobilization systems exemplify a growing trend in biocatalytic nanotechnology, with tailored surface chemistry and hybrid organic–inorganic matrices being used to optimize enzyme orientation, stability, and substrate accessibility. These strategies could not only improve the cost-effectiveness of enzymatic melanin degradation, but also open avenues for scalable and sustainable pigment-removal processes applicable in biomedical, cosmetic, and environmental remediation contexts. The immobilization of LiP onto MFO-GO hybrid supports provides a robust solution to the stability challenges often encountered in melanin processing. Specifically, these nanobiocatalysts exhibit enhanced resistance to the specific pH variations required to maintain melanin in a soluble state, preventing enzyme denaturation. Furthermore, the magnetic properties of the MFO core allow for rapid recovery and high reusability over multiple catalytic cycles, ensuring the long-term operational stability necessary for cost-effective industrial and cosmetic applications (Rathour et al. 2024).

### Degradation vs. Inhibition of synthesis

It is important to distinguish between the *degradation* of existing melanin polymers and the *inhibition* of their biosynthesis, as both processes can lead to pigment lightening but through fundamentally distinct mechanisms. Melanin degradation involves the breakdown or depolymerization of existing pigment structures, typically mediated by oxidative, enzymatic, or photochemical processes that dismantle the indole-quinone backbone. In contrast, inhibition of synthesis targets the upstream metabolic and enzymatic pathways responsible for melanin formation, resulting in reduced pigment production rather than destruction of existing material. For instance, Li et al. (2021) investigated *Auricularia auricula-judae*, a pheomelanin producer mushroom, and observed that freezing treatment led to noticeable lighter-colored fruiting bodies. In fact, this discoloration was not a result of melanin degradation, but a suppression of melanin biosynthesis (Li et al. 2021). The freezing-induced oxidative stress, enhanced the expression and activity of superoxide dismutase (SOD) and peroxidase (POD), leading to an altered intracellular redox balance that inhibited tyrosinase activity, key for the melanogenesis cascade. Additionally, freezing disrupted cysteine metabolism, critical pathway for a pheomelanin precursor formation, thereby reducing substrate availability for pigment synthesis, representing a metabolic, rather than an enzymatic approach to “decolorization.” (Li et al. 2021).

## Comparative analysis and applications

### Comparative analysis

A comparison of degradation methods reveals distinct advantages and limitations. To clarify these distinctions, Table 1 offers a comprehensive analysis between analytical chemical oxidation and applied enzymatic systems, summarizing the primary agents, underlying mechanisms, and inherent limitations of each methodology.

- *Mechanistic Depth*: Physical methods (UAE, MWE) primarily cause matrix disruption (Singh et al. 2021). Chemical methods (KMnO_4_, H_2_O_2_) are effective but harsh, nonspecific oxidants (Guo et al. 2023; Wakamatsu and Ito 2023). Biological methods are the most sophisticated, using highly specific (e.g., GPX) (Jeon et al. 2021) or potent, targeted shuttle mechanisms (e.g., LiP-VA, Lac-ABTS, MnP-Mn³⁺) (Khammuang and Sarnthima 2013; Kim et al. 2016a; Sadaqat et al. 2020).
- *Efficiency*: Biological systems enhanced by mediators show the highest efficiency. LiP-VA achieved 92% decolorization (Sadaqat et al. 2020), and Lac-ABTS reached 87% (Khammuang and Sarnthima 2013). Immobilization and complexing (e.g., LiP@MFO-Chit, Lac-DyP) significantly improve performance over free enzymes (Shin et al. 2019; Rathour et al. 2024).
- *Environmental Sustainability*: This is the primary driver for biological methods (Sadaqat et al. 2020). However, the toxicity of the *mediators* required for high efficiency is a major bottleneck. Both synthetic ABTS and the *natural* mediator veratryl alcohol (VA) are cytotoxic (Sadaqat et al. 2020). This highlights the need for safer, natural mediators like vanillin, which, although less efficient (45% decolorization), is a safer alternative (Khammuang and Sarnthima 2013). The synergistic complex (Shin et al. 2019) and the GPX system (Jeon et al. 2021) are promising as they rely on *internally generated* or recycled H_2_O_2_.
- *Substrate Specificity*: The reviewed studies successfully degraded both major types of melanin. DHN-melanin was oxidized by laccase-HBT and MnP (Tavzes et al. 2009). L-DOPA melanin was degraded by LiP-VA (Sadaqat et al. 2020), laccase-ABTS (Khammuang and Sarnthima 2013), the laccase-peroxidase complex (Shin et al. 2019), the GPX system (Jeon et al. 2021)d *fumigatus* (Luther and Lipke 1980).

**Table 1:** Summary of degradation strategies for microbial melanin: a comparative analysis between analytical chemical oxidation and applied biological enzymatic systems, including the role of oxidative mediators

Method	Primary agents	Mechanism	Efficiency	Mediator	Key limitation	Sources
Physical	Ultrasound, Microwave	Cavitation, Dielectric Heating	Extraction	N/A	Matrix disruption, not in situ	(Singh et al. 2021)
Chemical	KMnO_4_, H_2_O_2_, HI	Oxidation, Reduction	High (Analytical)	N/A	Not applied for in situ degradation	(Borges et al. 2001; Wakamatsu and Ito 2023)
Biological (LiP)	Lignin Peroxidase	Radical Cation (VA^•+^)	Very High (92%)	Veratryl Alcohol (VA)	Mediator toxicity	(Sadaqat et al. 2020; Rathour et al. 2024)
Biological (MnP)	Manganese Peroxidase	Mn^3+^ Oxidation	High	Mn^2+^	H_2_O_2_ required	(Tavzes et al. 2009; Kim et al. 2016a)
Biological (Lac)	Laccase	Radical Cation (ABTS, HBT)	High (87%)	ABTS, HBT, Vanillin	Mediator toxicity	(Tavzes et al. 2009; Khammuang and Sarnthima 2013)
Biological (Complex)	Lac-DyP Complex	H_2_O_2_ Recycling	High (6.7x Lac)	Internal (H_2_O_2_)	Complex engineering	(Shin et al. 2019)
Biological (Cellular)	*A. fumigatus*, *Geotrichum* sp.	Catabolism, Bioconversion	High (90%)	N/A	Slow, bioconversion	(Luther and Lipke 1980; Kim et al. 2016a)

### Applications

**Fig. 3:**
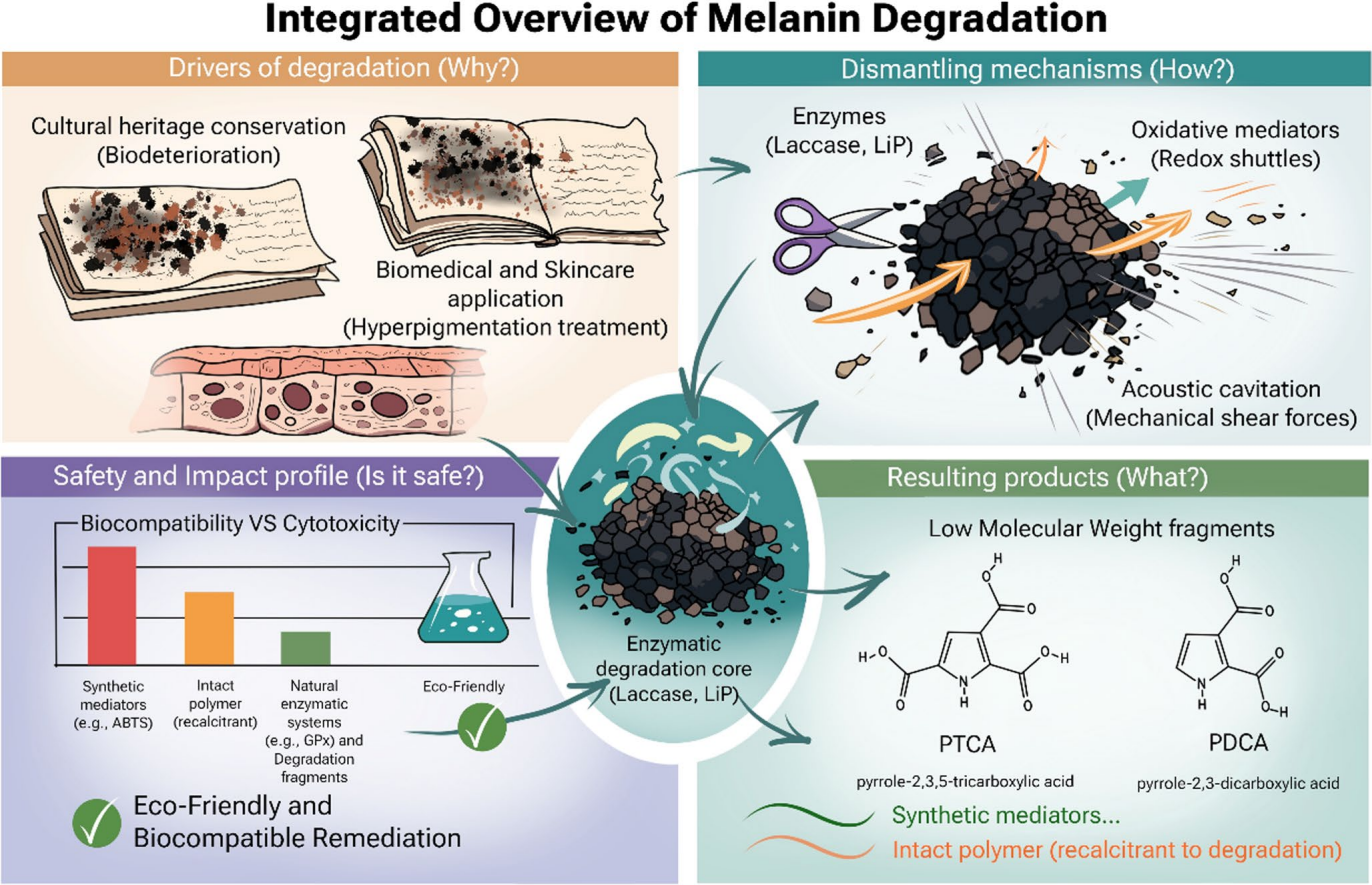
The diagram illustrates the complete degradation lifecycle, encompassing: Drivers of degradation, using biodeterioration and hyperpigmentation treatment as examples; Some mechanisms of dismantling the melanin, like enzymes, acoustic cavitation and oxidative mediators; Resulting chemical markers like PTCA and PDCA; and a comparative safety profile of catalysts and by-products

- *Biomedical and Cosmetic Applications*: This is the most prominent application, focusing on “skin whitening” or depigmentation (Shin et al. 2019; Sadaqat et al. 2020; Jeon et al. 2021; Rathour et al. 2024). Enzymatic approaches (LiP, MnP, GPX, Lac-DyP complex) are proposed as safer alternatives to cytotoxic tyrosinase inhibitors (Kim et al. 2016a; Sadaqat et al. 2020; Guo et al. 2023) (Fig. 3). Studies successfully demonstrated melanin reduction in human corneocytes (Shin et al. 2019) and 3D artificial skin models (Jeon et al. 2021).
- *Art Conservation*: The ability of laccase-HBT systems to bleach mold stains (DHN-melanin) from paper demonstrates a key application in the conservation of cultural heritage (Fig. 3) (Tavzes et al. 2009).
- *Industrial Pigment Modification / Food Preservation*: The antioxidant properties of fungal melanin are harnessed for industrial applications. Subramaniam et al. (2024) demonstrated that films made of gelatin/melanin or agar/melanin act as potent antioxidants, effectively delaying oxidative rancidification (measured by peroxide and acid values) in pork lard, thereby extending its shelf life during trade (Subramaniam et al. 2024).
- *Bioremediation*: Ligninolytic enzymes (laccase, peroxidase) from white-rot fungi are used to degrade synthetic dyes in industrial wastewater (Muñoz-Torres et al. 2024). Furthermore, the *binding* properties of melanin are used for the bioremediation of heavy metals (e.g., Cd, Cr, Pb, Cu) (Cordero and Casadevall 2017; Singh et al. 2021; Muñoz-Torres et al. 2024) and radionuclides (Eisenman and Casadevall 2012a; Guo et al. 2023).
- *Other Industrial Applications*: Melanin’s unique properties make it suitable for use as a biopesticide photoprotectant (Sansinenea and Ortiz 2015; Guo et al. 2023; Muñoz-Torres et al. 2024), a natural colorant (Chhoker et al. 2025), a component in nanotechnology (Singh et al. 2021; Rathour et al. 2024), and in bioelectronics (Singh et al. 2021).

## Discussion and concluding remarks

This comprehensive review illustrates that the degradation of microbial melanin, a biopolymer defined by its recalcitrance (Bell and Wheeler 1986; Guo et al. 2023; Chhoker et al. 2025), is a multifaceted challenge being addressed through chemical, physical, and biological lenses. A clear distinction has emerged between degradation for *analytical* purposes and degradation for *applied* purposes.

Analytical degradation, used to elucidate the complex, amorphous structure of melanin (Nosanchuk et al. 2015; Pralea et al. 2019; Wakamatsu and Ito 2023), relies on harsh chemical methods. These include KMnO_4_ or alkaline H_2_O_2_ oxidation and HI hydrolysis (Borges et al. 2001; Guo et al. 2023; Wakamatsu and Ito 2023). These methods effectively break the polymer into its identifiable monomeric markers (e.g., PTCA, PDCA, AHP, 3AT) (Borges et al. 2001; Wakamatsu and Ito 2023), providing the “gold standard” for quantifying eumelanin versus pheomelanin content in tissues (Borges et al. 2001; Simon and Peles 2010).

For applied degradation (e.g., cosmetics, bioremediation), biological methods are unequivocally favored for their potential specificity and sustainability (Kim et al. 2016a; Sadaqat et al. 2020). The consensus across studies (Tavzes et al. 2009; Eisenman and Casadevall 2012a; Kim et al. 2016a; Sadaqat et al. 2020) is that the ligninolytic enzymatic machinery of white-rot fungi (LiP, MnP, and laccases) is the most effective tool, treating melanin as an analogous substrate to lignin (Tavzes et al. 2009; Rathour et al. 2024).

The structural similarity between microbial and human eumelanin allows for the cross-application of degradation strategies. Enzymatic systems derived from microorganisms, such as the laccase-peroxidase complexes and LiP systems discussed herein, have already demonstrated efficacy in human corneocytes and 3D skin models, reinforcing that microbial melanin research is a critical driver for the next generation of natural human skin-whitening products (Eisenman and Casadevall 2012a; Pillaiyar et al. 2017).

A critical finding is that direct enzymatic oxidation is often inefficient. High degradation rates almost exclusively rely on *mediators* (Tavzes et al. 2009; Khammuang and Sarnthima 2013; Sadaqat et al. 2020). These mediators (e.g., VA, ABTS, HBT, Mn^2+^) act as diffusible “oxidative shuttles” that can penetrate the complex, amorphous structure of the melanin polymer (Khammuang and Sarnthima 2013; Nosanchuk et al. 2015; Sadaqat et al. 2020). This reliance reveals the primary bottleneck for biological methods: toxicity. The high cytotoxicity of veratryl alcohol (VA), even as a natural product, renders its direct application in cosmetics problematic (Sadaqat et al. 2020). This identifies a major research gap: the discovery or engineering of safe, highly efficient mediators. The synergistic complex developed by Shin et al. (2019) (Shin et al. 2019) and the lysosome/GPX system (Jeon et al. 2021) represent significant innovations, as they utilize internally generated or recycled H_2_O_2_ and bypass the need for external, toxic mediators. Furthermore, biotechnological strategies such as immobilizing enzymes on nanoparticles (Rathour et al. 2024) enhance stability, reusability, and targeted delivery, making industrial application more feasible.

To provide a comprehensive synthesis of the degradation process, Fig. 3 offers an integrated overview of the melanin dismantling lifecycle, connecting some examples of industrial and clinical drivers to the biochemical mechanisms, resulting markers, and respective safety profiles. An essential question for the biotechnological application of melanin degradation is whether the process compromises the pigment’s inherent radical scavenging activity. While enzymatic oxidation and subsequent decolorization involve the dismantling of the polymer’s conjugated backbone, the resulting low-molecular-weight fragments, such as pyrrole-based acids (PTCA/PDCA) or phenolic derivatives, may retain significant antioxidant potential. In fact, the biological activity of these fragments could provide a secondary benefit in cosmetic formulations, where depigmentation is ideally coupled with continued oxidative protection. However, evidence from extreme halotolerant models like *H. werneckii* indicates that intracellular systems, rather than the melanin polymer itself, may be the primary defense against certain oxidants like H_2_O_2_, (Kejžar et al. 2013; Rani et al. 2013), suggesting that the functional impact of degradation products remains a fertile ground for future investigation.

This review also uncovered an alternative pathway to depigmentation, highlighted by Li et al. (2021): achieving a lighter color not by degrading the polymer, but by inhibiting its synthesis (Li et al. 2021). Freezing treatment on *A. auricula-judae* induced oxidative stress that inhibited tyrosinase and disrupted the cysteine metabolism pathway, thereby reducing the production of pheomelanin (Li et al. 2021). This illustrates a metabolic control mechanism distinct from external degradation.

Finally, significant research gaps remain. The literature on *physical* degradation methods (e.g., ultrasound, microwave) is limited almost exclusively to extraction, not in situ degradation (Singh et al. 2021). Furthermore, while the phototoxicity of pheomelanin is documented (Simon and Peles 2010), its exploitation for selective photodegradation is unexplored. Most degradation studies focus on DOPA-eumelanin or DHN-allomelanin; the specific enzymatic degradation of sulfur-containing pheomelanin is rarely addressed. A crucial next step will be to link the known physical nanostructure of melanin—the granules, stacked sheets, and pores (Nosanchuk et al. 2015; Chhoker et al. 2025) and the specific mechanisms of enzymatic and mediator attack. Moving beyond the established “gold standard” chemical methods is essential for sustainable application. Linking physical nanostructure to specific enzymatic and mediator mechanisms remains the most crucial next step for applications in medicine, industry, and environmental remediation.

## Data Availability

No datasets were generated or analysed during the current study.
